# Comparison between two portal laparoscopy and open surgery for ovariectomy in dogs

**Published:** 2014

**Authors:** Elnaz Shariati, Jalal Bakhtiari, Alireza Khalaj, Amir Niasari-Naslaji

**Affiliations:** 1 *Department of Surgery and Radiology, Faculty of Veterinary Medicine, University of Tehran, Tehran, Iran; *; 2 *Department of surgery, Faculty of Medicine, Shahed University, Tehran, Iran.*

**Keywords:** Dog, Laparoscopy, Ovariectomy, Two Portal

## Abstract

Ovariectomy (OVE) is a routine surgical procedure for neutering in small animal practice. Laparoscopy is a new surgical technique which contains advantages such as less trauma, smaller incision and excellent visualization than traditional open surgery. The present study was conducted to examine the feasibility and safety of laparoscopic procedure through two portal comparing with the conventional open surgery for OVE in healthy female bitches (n=16). Dogs were divided in two equal groups. In laparoscopic group, two 5 and 10 mm portals were inserted; First in the umbilicus for introducing the camera and the second, caudal to the umbilicus for inserting the forceps. Laparoscopic procedure involved grasping and tacking the ovary to the abdominal wall, followed by electrocautery, resection and removal of the ovary. In open surgery, routine OVE was conducted through an incision from umbilicus to caudal midline. Mean operative time, total length of scar, blood loss, clinical and blood parameters and all intra and post-operative complications were recorded in both groups. Mean operative time, total length of scar, blood loss and post-operative adhesions were significantly less in laparoscopic group compared with open surgery. In conclusion, laparoscopic OVE is an acceptable procedure due to more advantages in comparison with traditional OVE.

## Introduction

Sterilization, also called neutering, is a surgical technique used to control sexual behavior and conception aiming at the prevention of uterine diseases such as pyometra and mammary gland tumor in small animals. This is achieved by either ovariohysterectomy (OHE) or ovariectomy (OVE).^1^ OVE has been accepted as a standard procedure and an alternative to OHE in Europe.^2^ Many advantages including reduced surgical time, smaller incision and fewer traumas have been reported for OVE.^3^ Laparoscopic approach for OVE has several advantages including prompt recovery, shorter anesthetic period, fewer trauma, less hemorrhage, and excellent visualization than traditional open surgery.^4^ The first laparoscopic sterilization of dogs was reported in 1985 by Wildt and Leowier.^5^ Since then laparoscopic surgery has become wide-spread as an alternative to open surgery due to its less invasiveness and better visualization.^4^^,^^6^^,^^7^ The objective of this study was to examine the feasibility and safety of two portal laparoscopic OVE and to compare this technique with the conventional OVE via midline open surgery in dog.

## Materials and Methods

The present study was approved by the Animal Ethics Committee of the Faculty of Veterinary Medicine, University of Tehran, Tehran, Iran. The experiment was conducted at Small Animal Veterinary Teaching Hospital, Faculty of Veterinary Medicine, University of Tehran on 16 adult female mixed breed dogs, weighting 14.0 ± 4.0 kg, with 12 to 16 months age. The dogs were divided into two equal groups, randomly. Food was restricted for 8 hr prior to surgery, and cefazolin (20 mg kg^-1^, IV; Jaber Ebne Hayyan, Tehran, Iran) was administrated as a preoperative pro-phylaxis at the time of inducing anesthesia. Dogs were sedated with acepromazine (0.05 mg kg^-1^, IM; Alfasan, Woerden, The Netherlands) and ketamine (10 mg kg^-1^, IM; Alfasan, Woerden, The Netherlands) and general anesthesia was induced by combination of ketamine (5 mg kg^-1^, IV) and diazepam (0.2 mg kg^-1^, IV; Chemi Darou Industrial Co., Tehran, Iran) and maintained under inhalation of iso-flurane (Baxter, Deerfield, USA) in 1.5% oxygen through anesthetic machine.

**Laparoscopic ovariectomy. **Dogs were placed in dorsal recumbency and reverse Trendelenburg position and the area from xiphoid to pubis was prepared under aseptic conditions. For laparoscopy, a 10 mm incision was made 1 to 2 cm caudal to umbilicus through the skin and sub-cutaneous tissue down to the linea alba. The linea alba was cut precisely for inserting the trocar into the abdomen under direct vision. Then the primary trocar was inserted, while ventral abdominal wall was pulled up to avoid trauma to visceral organs. The surgical time was started at this point. Then pneumoperitoneum was established by connecting the trocar to the high flow insufflators (Richard Wolf, Knittlingen, Germany) using carbon dioxide until the pressure of 12 mm Hg was achieved. The 10 mm 0 degree rigid camera (Karl Storz, Tuttlingen, Germany) connected to a light source was inserted into the abdomen and a 360 degree scan was performed to check for any existing abnormalities. A 5 mm skin incision was made midway between the umbilicus and pubis and the second 5 mm portal was inserted under direct visualization to prevent injury to abdominal organs. Then, the dogs were tilted 30 degrees either to the right or left lateral recumbency to perform left or right OVE, respectively.

**Fig. 1:**
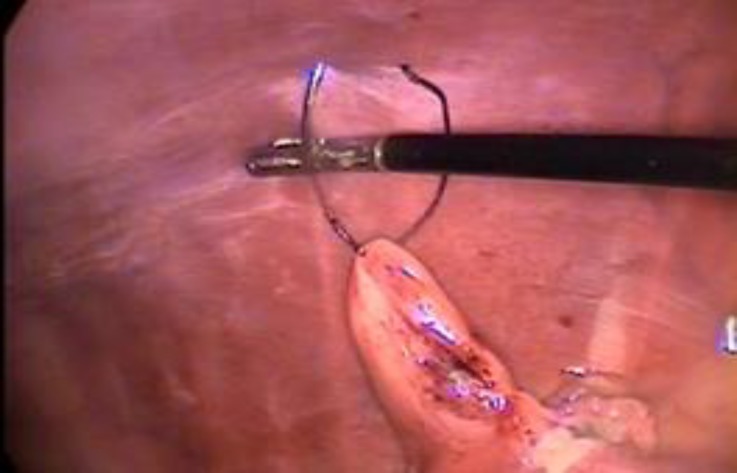
The ovary was tacked to the body wall by passing a 5 cm, 3/8 circle curved cutting needle

**Fig. 2:**
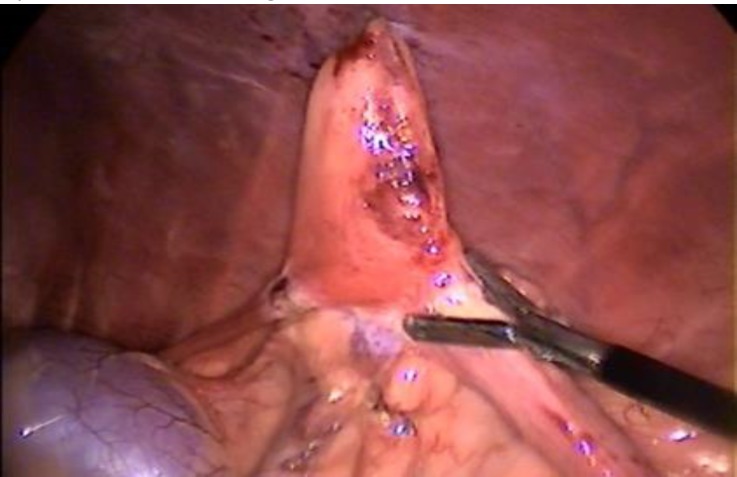
The ovarian pedicle, proper ligament and mesovarium was cauterized with bipolar electrocautery

For OVE, the left or right kidney was identified as a land mark. Then proper ligament of the ovary was grasped by the 5 mm grasping forceps and then elevated and tacked to the body wall by passing a 5 cm, 3/8 circle curved cutting needle and sutured percutaneously through the body wall (Fig. 1). Then, the 5 mm bipolar electro-cautery forceps was introduced via the caudal portal and the ovarian pedicle, proper ligament and mesovarium were cauterized (Fig. 2). Following ensuring hemostasis of the ovarian pedicle, the 5 mm laparoscopic scissors was inserted into abdomen from the caudal portal to resect the ovary. At the end of the procedure, the grasping forceps holding the resected ovary, was brought directly towards the camera while the camera was simultaneously pulled backwards until the forceps entered inside the cannula and was observed from the outside. Then, the cannula was pulled out and the ovary was removed from the abdomen. The cannula was re-inserted and the patient was tilted 30˚ to the other side to perform OVE on the contralateral side. The same procedure was repeated. After removing the ovaries, the abdomen was scanned for ensuring hemo-stasis or any other complications. The portal sites were sutured in 2 simple interrupted layers using 0 polyglycolic acid (Teb Keyhan, Eshtehard, Iran) for inner layers and 2/0 nylon (Teb Keyhan, Eshtehard, Iran) for skin.

**Open ovariectomy. **In the conventional open OVE, a 4 to 6 cm ventral midline skin incision was performed starting from the umbilicus and extended caudally. The ovaries were identified following either the left or right uterine horn proximally. Simple and transfixating ligatures were used both over the ovarian pedicle and at the location of proper ligament of ovary to close the uterine horn with the 0 polyglycolic acid. Ovaries were resected and the pedicle was checked for hemorrhage. The uterine horn was released into the abdomen. The procedure was repeated for the second ovary and finally the abdominal incision was closed in a routine three layers manner using the 0 polyglycolic acid for inner layers in simple continuous pattern and the 2/0 nylon for skin in simple interrupted pattern. The surgical time was consisted the duration between skin incision and end of closure.

Mean operative time, estimated blood loss, incision length, intra and post-operative complications were recorded in both groups. Blood loss was estimated by the collected blood volume using suction in laparoscopy and saturated gauze sponges in open surgery. Complete blood count (CBC) and clinical signs including heart rate, respiratory rate and body temperature were also measured on days 0, 1, 3 and 7 after surgery in both groups. The surgical wounds were evaluated for any complications every day (3 to 5 days). Two weeks following the surgery, sutures were removed under general anesthesia and dorsal recumbency position, and the skin was incised at the umbilicus and the 10 mm trocar inserted. Pnuemo-peritonuem was achieved and the camera inserted for evaluation of intra-abdominal adhesions in both groups.

**Statistical Analysis. **Data with continuous nature such as operative time and length of surgical scar were analyzed using Student *t*-test if assumptions of parametric tests were fulfilled otherwise they were analyzed using Kruskal-Wallis test. Data with frequency nature such as heart rate and respiratory rate were analyzed using Chi-Square test in SAS (Version 8.2; SAS Institute, Carry, USA). Data were presented as mean ± SEM.

## Results

All dogs in both groups were recovered uneventfully and there was no need to convert the surgeries to open in laparoscopic group. All clinical findings include heart rate, respiratory rate, body temperature and blood parameters were within normal ranges (*p *> 0.05), (Tables 1 and 2). 

**Table 1:** Vital signs in days 0, 1, 3, and 7 after ovariectomy in laparoscopic and open surgery

**Group**	**Day**	**Body temp.** **(** **˚** **C)**	**Respiratory rate (bpm** * **) **	**Heart rate (bpm)**
**Laparoscopic surgery**	0	39.12 ± 0.17	26.37 ± 0.61	96.25 ± 1.94
	1	39.50 ± 0.12	28.12 ± 1.00	86.87 ± 2.21
	3	39.06 ± 0.18	28.37 ± 1.02	108.12 ± 4.14
	7	38.93 ± 0.22	29.12 ± 0.94	98.25 ± 2.03
**Open surgery**	0	39.50 ± 0.12	27.25 ± 1.14	100.50 ± 2.37
	1	38.80 ± 0.26	24.12 ± 1.02	94.50 ± 1.47
	3	39.00 ± 0.19	26.50 ± 0.63	90.00 ± 1.87
	7	38.62 ± 0.26	28.00 ± 1.19	94.75 ± 1.37

* bpm: breath/beat per min.

**Fig. 3:**
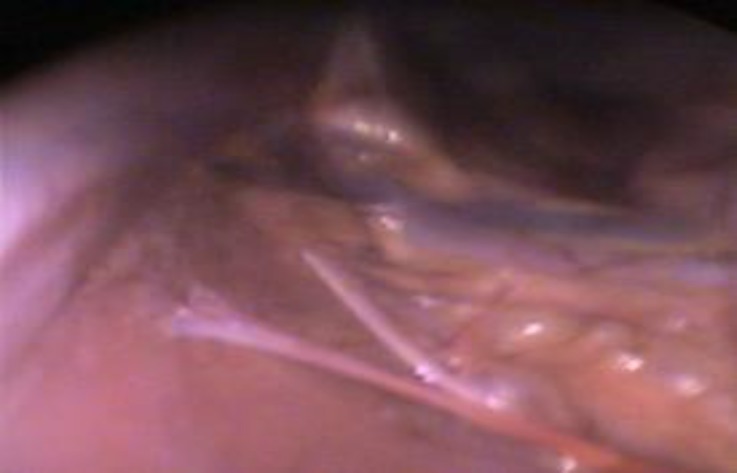
Adhesion of ovarian pedicle to small intestine in laparoscopy group

**Fig. 4:**
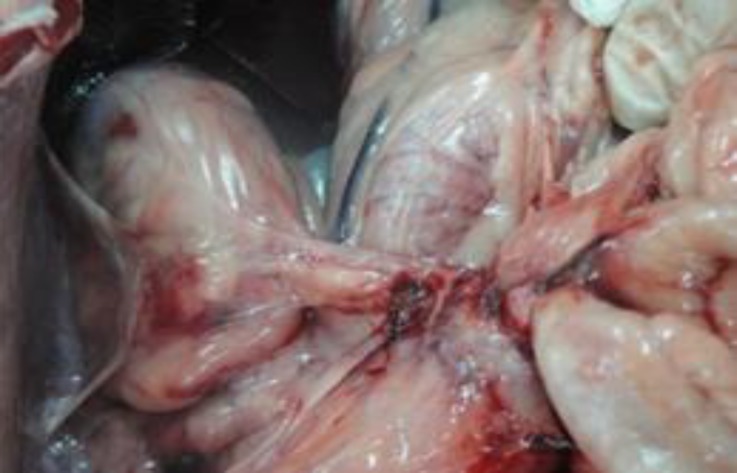
Adhesion of omentum to abdominal wall in open surgery group

Operative times were 17.7 ± 1.2 and 36.6 ± 1.6 min in laparoscopy and conventional methods, respectively (*p* < 0.05). Blood loss was less in laparoscopy (< 2 mL) than in conventional approach (< 8 mL; *p *< 0.05). Total length of surgical scar was longer in conventional group (54.0 ± 10.0 mm) compared to laparoscopy group (17.0 ± 2.0 mm; *p* < 0.05). No intra-operative complications were occurred in both groups. Visualization of the ovarian tissue was excellent in laparoscopy. Wound complications including hernia formation, hematoma or infection did not occur in any dog in both groups. More post-operative adhesions were occurred in conventional method compared to laparoscopy, with higher incidence around the ovarian pedicle (Table 3, Figs. 3 and 4).

**Table 2:** Blood parameters in days 0, 1, 3, and 7 after ovariectomy in laparoscopic and open surgery

**Group**	**Day**	**PCV** * **(%)**	**Hemoglobin** **(g dL** ^-1^ **)**	**WBC** * **(10** ^3^ ** µL** ^-1^ **)**	**Neutrophil** **(%)**	**Band cell** **(%)**	**Lymphocyte** **(%)**	**Eosinophil** **(%)**	**Monocyte** **(%)**
**Laparoscopic surgery**	0	39.30 ± 3.06	14.70 ± 0.78	14.20 ± 0.85	78.00 ± 1.78	1.00 ± 0.25	18.75 ± 1.16	-	2.00 ± 0.35
	1	47.20 ± 3.47	11.90 ± 3.42	17.10 ± 3.12	76.00 ± 1.10	2.00 ± 0.50	19.62 ± 1.19	1.00 ± 0.30	4.00 ± 0.43
	3	44.60 ± 2.53	10.30 ± 2.53	15.90 ± 1.56	69.00 ± 0.98	1.00 ± 0.25	28.00 ± 1.17	2.00 ± 0.35	2.00 ± 0.39
	7	51.20 ± 3.03	14.40 ± 2.97	15.20 ± 2.97	66.00 ± 1.17	-	30.00 ± 1.14	1.00 ± 0.35	3.00 ± 0.43
**Open surgery**	0	41.60 ± 5.11	15.60 ± 3.61	15.50 ± 1.23	71.00 ± 0.79	2.00 ± 0.35	16.62 ± 1.31	1.00 ± 0.25	2.00 ± 0.50
	1	37.50 ± 2.48	13.20 ± 1.77	20.80 ± 1.03	80.00 ± 1.48	3.00 ± 0.39	10.75 ± 0.57	3.00 ± 0.25	3.00 ± 0.30
	3	45.90 ± 3.07	10.50 ± 0.95	19.70 ± 3.36	77.00 ± 1.56	3.00 ± 0.46	12.87 ± 0.67	1.00 ± 0.30	2.00 ± 0.43
	7	53.70 ± 2.88	11.70 ± 1.98	17.10 ± 0.78	69.00 ± 1.02	2.00 ± 0.50	16.50 ± 1.40	1.00 ± 0.35	2.00 ± 0.50

* PCV: Packed cell volume, WBC: White blood cells.

**Table 3:** Frequency and location of adhesions observed two weeks after ovariectomy by laparoscopic or conventional open surgery

**Group **	**Location of adhesion**	**Frequency**
**Laparoscopic ** **ovariectomy**	Omentum to abdominal wall Ovarian pedicle to capsule of kidney	1 1
**Conventional ** **ovariectomy**	Omentum to incision line Ovarian pedicle to small intestine Ovarian pedicle to abdominal wall Ovarian pedicle to omentum	3 1 2 2

## Discussion

In the last few years, the use of laparoscopy in veterinary medicine has expanded and consequently establishing the advantages, disadvantages and possible complications of each procedure is needed. Shorter hospital stays, decreased postoperative pain, rapid return to preoperative activity, decreased postoperative ileus, and preserved immune function are among the benefits of the laparoscopic approach. However, the instruments cost of laparoscopy afford, surgeon’s limited precision, poor ergonomy, and shortage of learning curve are probable disadvantages to be considered.^8^

Bilateral OVE with open surgery or laparoscopy is a method of choice for neutering of the bitches.^4^^,^^9^ Because of numerous advantages of laparoscopic procedure, it has become an alternative to traditional midline OHE.^10^ Bakhtiari *et al.* performed the first laparoscopic canine neutering procedure in Iran.^11^ Nowadays laparoscopic OVE is the method of choice to develop neutering technique.^12^

Variety of methods for laparoscopic OVE with different number of portals have been described in veterinary medicine.^13^ In order to enhance the feasibility and cosmetics of the techniques, two portals rather than three portals were chosen in the present study, as it was recommended previously.^14^

In present study the surgical time in laparoscopic procedure was significantly less than conventional method. The duration of laparoscopy was shorter than the one reported previously (30 min), while the open operative time was similar in the same study (60 min).^15^ In another report the mean surgical time by two portal laparoscopic procedure was 19.6 min which was similar to our findings.^16^ In contrast to previously reported studies, which rejected laparoscopic surgery because of its prolonged duration of surgery compared with traditional midline OVE, we observed that surgical time could be compatible.^17^ The differences between duration of operative time in different studies can be influenced by surgeons experiences, body condition score which is related to amount of fat around the ovarian pedicle, body weight, position of the patient and vessel sealing devices.^18^^,^^19^

Total length of incision in laparoscopic group was significantly shorter than conventional one. Length of scars of laparoscopic surgeries was similar to the results reported by Dupre *et al*. (5 mm and 10 mm)^16^ but more than that reported by Culp *et al*. (3.5 mm and 6 mm),^20^ however, all of them were less than the open procedure.

Hemorrhage is one of the most common complications following OVE, occurring at the ovarian pedicles and broad ligaments. In this study, hemorrhage was also less in laparo-scopy compared to conventional group. The laparoscopic procedure provided better visualization of the abdomen cavity and vessels and therefore less hemorrhage occurred due to less manipulation and better coagulation because of bipolar hemostatic system which is based on electrical energy and able to seal vessels with a diameter up to 7 mm.^21^ Similar results concerning the advantages of laparoscopy was also reported by Austin *et al*.^6^

The extent of adhesions after open surgery was significantly higher than laparoscopy due to longer incision, application of suture material, more manipulation resulting in more traumas and more hemorrhage. Schippers reported extensive adhesion formation after canine bowel resection following laparotomy compared with laparoscopy.^22^

In conclusion, laparoscopic OVE is an easy, safe and acceptable procedure due to its various advantages which is recommended instead of traditional open surgery OVE from midline laparotomy.
